# Investigating the Influence of Probe Pressure on Human Skin Using Diffusive Reflection Spectroscopy

**DOI:** 10.3390/mi14101955

**Published:** 2023-10-20

**Authors:** Israr Ahmed, Murad Ali, Haider Butt

**Affiliations:** Department of Mechanical and Materials Engineering, Khalifa University, Abu Dhabi 127788, United Arab Emirates; murad.ali@ku.ac.ae

**Keywords:** diffusive reflection spectroscopy, pressure probe, optical fiber, dermal studies, hemoglobin

## Abstract

The skin has emerge as a compelling subject for investigation owing to its accessibility and the relatively straightforward application of optical procedures to it. Diffusive reflection spectroscopy (DRS) was employed to study the influence of probe pressure on human skin. A comprehensive non-invasive study was conducted, which covers almost all the important body parts for in vivo measurements. Reflection spectra were measured for the fingertip, forearm, forehead, neck, and foot under a set of probe pressures (0–265 kPa). Importantly, each tissue type’s unique composition and morphology influenced the shape, size, intensity, and position of the recorded peak, highlighting the tissue-specific responses to pressure. In addition, time-based reflection spectroscopy was also performed on the forearm under blood occlusion for 5 min to study the effect. DRS measurements were performed on volunteers of different skin tones, including dark, medium, and fair. Later, a change in the intensity of the oxyhemoglobin peak was confirmed using a green laser light of a wavelength of 532 nm. Besides the dermal studies, diffusive reflection spectroscopy was also employed to investigate the probe pressure effect on human nails. A probe pressure ranging from 0 to 385 kPa was applied for nail spectroscopy. The same trend of intensity change was observed following the previous measurements. The suggested sensing system may be crucial in applications requiring pressure sensing when the human body is subjected to varying pressures, such as exercise, weightlifting, and other sports.

## 1. Introduction

Skin is a multifaceted organ with diverse functions, and its pivotal role in dermal studies extends beyond its protective function. Investigating the skin’s structure, function, and response to various stimuli is essential for advancing medical science, healthcare, cosmetic innovation, and our overall understanding of the human body. The use of optical-fiber sensing technology has become prevalent for non-invasive sensing applications because of its simplicity and ease of operation [1,2,3,4,5]. The demand for sensors based on the reflection principle has increased for wearable devices, where the continuous monitoring of oxyhemoglobin levels is required, especially for sports activities. For sportswear, it is extremely important to know the oxygen saturation levels under specific pressures and the knowledge of how much pressure/load a human body can bear during any sports activity. To understand the origin of many diseases like diabetic neuropathy and venous ulceration, the knowledge of the oxygen saturation level of the blood is essential [6].

Diffusive reflection spectroscopy (DRS) is an optical technique employed for the non-invasive analysis of the optical characteristics of various materials, including biological tissues such as skin [7]. The phenomenon under consideration is grounded in the fundamental concept of diffusive reflection, wherein light interacts with a material and disperses in multiple directions as a result of its interactions with the microstructure and constituents of that material. The scattered light contains valuable data about the material’s composition, structure, and various optical properties. The DRS is based on the collection of this scattered light and subsequent spectrum analysis of diffusely reflected light using a spectrometer [2,8]. It is one of the most important tools for measuring the oxyhemoglobin level in human skin and is divided into two approaches [8,9,10,11,12,13,14,15,16]. In the first in vivo approach, an integrating sphere collects all the reflected light from the examination site, which is converted to a specific signal [17]. On the other hand, the second approach is very simple in operation, where an optical-fiber probe is introduced to the skin site, and the reflection signal is recorded using a spectrometer/power meter. This approach is preferred for clinical operations because of its simplicity and fast response. An optical fiber can be pressed against the skin, which can change the signal response under different applied pressures, as reported by many researchers [9,11,12].

The DRS has been reported extensively in the literature for dermal studies. In 2004, Savaasand et al., reported the effect of a pressure cuff applied to the upper arm and hand of human volunteers [18]. A set of pressures ranging from 80–100 mm Hg was applied for a duration of 5 min. Reflection measurements were carried out before and after administering the pressure cuff in the visible wavelength range of 450–800 nm. The change in the reflection spectra was recorded for increasing pressure values. This change was due to a change in blood volume fraction under the influence of the pressure cuff. In 2005, Randeberg et al. reported diffusive reflection spectroscopy measured in the visible wavelength range of 450–850 nm. A series of pressures were applied against the skin of human volunteers ranging from 70 to 150 kPa. The measurement setup includes a hand-held integrated sphere, making the applied pressure easier. The increase in the reflection spectrum was observed with increasing pressure along with the flattering of the oxyhemoglobin signature peak [17]. 

A DRS study for discrimination between early melanoma and dysplastic nevus was reported by Murphy et al. in 2005 [19]. The experimental setup comprised an optical-fiber system for investigating pigmented lesions. DRS spectra were collected from 120 pigmented lesions, including 64 histopathologically diagnosed melanoma. The variations in spectra between different diagnoses were examined for discriminant analysis [19]. Another DRS study on six male rats was reported by Yalina and Wei-Chiang et al. in 2008. The spectroscopic system comprising optical fiber was utilized for in vivo DRS measurements. A set of probe pressures (0, 25.8, 32.5, 40.6, and 48 kPa) were applied to the liver and heart tissue of the rats. An increase in the reflection spectra was observed for all measurements under the influence of applied pressure [20]. Similarly, Reif et al. in 2008 reported a DRS study applied to the thigh of mice. Ten mice were used for this experiment, and the thigh skin was removed to expose the thigh muscles. An optical-fiber system was used to carry out the measurements by pressing the probe against the thigh muscles of mice. A probe pressure of 4, 9, 13, and 20 kPa was applied, and reflection spectra were obtained, confirming the change in the optical properties of tissue under applied pressure [21].

The reflection measurement of human skin was reported in 2009 by Delgado. This study involved 45 adult volunteers. A set of probe pressures of 2.02, 3.88, 5.76, 7.87, and 9.33 kPa were applied to the forearm of all volunteers. An increase in reflection spectra was observed for all the measurements with increasing pressure. Within the visible wavelengths of 540 to 570 nm, a “W” shape trend was observed in the spectrum, which is the signature peak of oxyhemoglobin, and with increasing pressure, this peak started vanishing as the blood volume was pulled out because of the probe pressure. It was also observed that the trend around 600 nm was not consistent for all volunteers [11]. In 2017, Alexey reported the influence of probe pressure on the human skin. These experiments were performed using a wavelength range of 200–1000 nm. The pressure was applied to the middle figure pad of five volunteers. A set of pressure ranging from 0 to 40 kPa was used. An increase in the reflection intensity of the oxyhemoglobin peak (525 to 575 nm) was observed for all measurements [9].

This study presents a non-invasive comprehensive investigation of the dermal characteristics of human skin. The study includes the spectroscopy analysis of the fingertips, forearm, forehead, and foot. Besides dermal studies, diffusive reflection spectroscopy was also applied to the human nail to record the response. This research was centered around the optical range of 450–750 nm, which has the capability to penetrate the capillary layer of the dermis. This capability enables the measurement of oxygen partial pressure in shallow areas such as nail folds. The utilization of this detection method holds potential for clinical applications in the diagnosis of hypoxia. Cardiovascular illness is typically identified as the primary etiological factor contributing to the occurrence of hypoxia [22]. Subsequently, the results will be discussed in the framework of pressure influencing the oxyhemoglobin level by changing the blood content. In short, this research employs a simple measurement system to comprehensively investigate various human body parts under pressure, utilizing diffusive reflection spectroscopy (DRS) spectra. The reflection spectra serve as an indicator to determine the pressure tolerance of human tissue, as evidenced by the conversion from the oxyhemoglobin peak to the deoxyhemoglobin peak.

## 2. Materials and Methods

**Figure 1:**
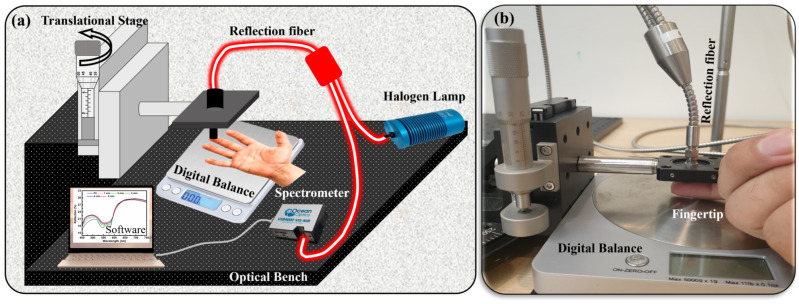
(**a**) Schematic of pressure-probe diffusive reflection setup and (**b**) finger under a pressure probe for reflection measurements.

An in-house self-made setup was used to conduct these dermal studies. The setup includes the reflection optical-fiber probe (RP21, Ø200 μm, 0.22 NA, Thorlabs, Inc., Newton, NJ, USA), Oceanview UV-vis spectrometer USB4000, Oceanview software, digital balance (for pressure measurement), halogen lamp (white light source), and translational stage connected to the micrometer accuracy in the z-direction attached to the optical fiber. The pressure was applied using this translational stage system (Figure 1a,b). The introduction of a translational stage played a pivotal role in the process, serving to guarantee both the accuracy of pressure application and the precise control over the pressure magnitude due to micrometer accuracy. A digital balance was used to measure the weight of the finger and hand for each experiment. A pressure formula was used for the conversion of weight into pressure. For example, for finger measurements, the weight of a finger was measured and set as the reference value in the presence of no pressure (P0). The schematic of the experimental setup can be seen in Figure 1a. After subtracting the finger mass, the remaining weight was converted to pressure. Various pressure levels were applied to different body parts ranging from P1 to P6, and are presented in Table 1. A reflection bundle optical fiber was used to collect and transmit the reflected signal from the examination site to the UV-vis spectrometer. The reflection spectra were acquired using Oceanview software in the visible wavelength range spanning from 450 nm to 750 nm. The integration time was set to automatic, resulting in the acquisition of an average of 20 scans for each spectrum. A white diffusive surface was used as a reference for all these spectral measurements. The experimental data evaluation was carried out using the Origin Pro software.

A green laser light of a wavelength of 532 nm was also employed to confirm the results obtained using white light. The laser measurement system was composed of a green laser (532 nm, 4.5 mW, Thorlabs, Inc., Newton, NJ, USA), a reflection optical-fiber probe (RP21, Ø200 μm, 0.22 NA, Thorlabs, Inc., Newton, NJ, USA), an optical power meter (PM100D, optical power 100 pW–200 W, Thorlabs, Inc., Newton, NJ, USA), and photodetectors (S120C, 400–1100 nm, 50 mW, Thorlabs, Inc., Newton, NJ, USA).

This study utilized diffused reflection spectroscopy on human skin to investigate the impact of applied pressure. Various pressures were exerted on different anatomical regions, such as the neck, forehead, forearm, fingertips, foot, and nails. The measurements were conducted on six volunteers with varying skin tones, encompassing individuals with dark, fair, and medium complexions. The experiment was repeated with different light sources, including white light and green laser. In the tests involving a green laser, the spectrometer was substituted with a photodetector that was connected to the power meter to measure the power intensity of the reflected light. The readings were recorded manually against every pressure value and then plotted accordingly. Hence, experimental data were collected from the fingers of both hands and the nails of all six volunteers.

Owing to the complexities involved in measuring applied pressures, except for those on the fingers and nails, the exact pressure values for the neck, forehead, forearm, and foot measurements could not be ascertained. Consequently, the probe was placed directly on the skin surface, with gradually increasing pressure. Additionally, an alternative method of blood occlusion overtime was utilized for forearm measurements. A controlled pressure cuff was gently administered to the upper arm, strategically designed to attenuate blood flow to the lower arm while prioritizing the volunteers’ comfort and safety. The precise adjustment of pressure levels ensured that it remained well within a tolerable range, mitigating any potential complications. Comprehensive data collection efforts were employed, encompassing a cohort of volunteers. Measurements were taken for all volunteers, and reflection spectra were recorded for 5 min with a time interval of 1 min for each reading.

## 3. Results

**Figure 2:**
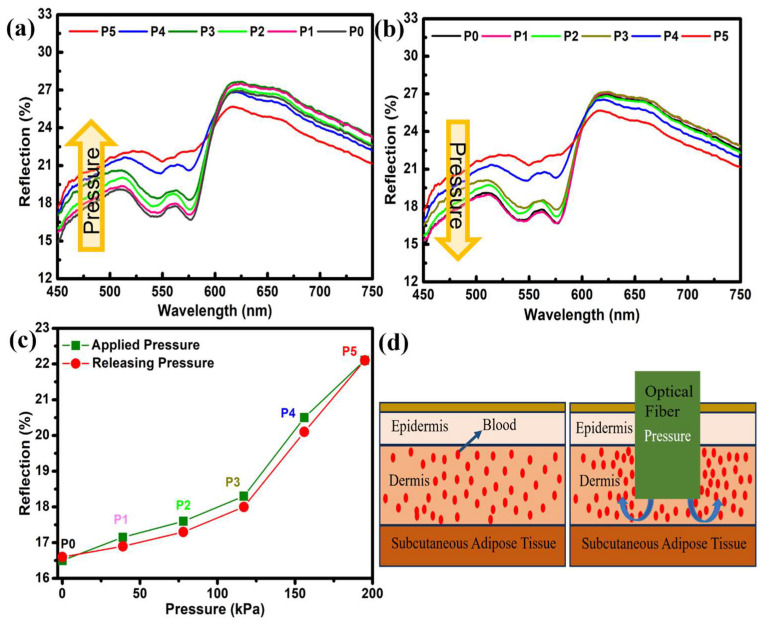
Reflection spectra of the finger under different pressures. (**a**) Applied pressure and (**b**) releasing pressure. (**c**) Quantitative analysis of reflection spectra under applied and released pressure. (**d**) Schematics of the skin layer and effect of applied pressure.

Pressure-based DRS was employed for dermal studies focusing on anatomical sites, including the fingertips, foot, neck, forehead, and forearm. The study started with the measurements taken for the fingertips. The DRS spectrum of the fingertip is illustrated in Figure 1a,b. The first spectrum was recorded under no pressure and termed P0. The black curve in Figure 1a,b shows the reflection spectra of the fingertip under normal conditions (no pressure). The spectra show a “W”-shape trend in the 500–600 nm wavelength range. This specific wavelength region is known for absorbing oxyhemoglobin in human blood. Therefore, the “W”-shape trend in reflection spectra is visible in this region. Later, the pressure was increased to P1, P2, … P5 (39–195 kPa). With increasing pressure, the change in the overall spectra, especially in the 500–600 nm region, can easily be seen. A 6% increase in the reflection spectra was observed, where reflection intensity increased from 16% to 22%. With increasing pressure, the flattering of the “W” shape can also be observed, which was prominent for P5. The calculated values of pressure are presented in Table 1. At 600 nm, a pivot point can be seen, after which the reflection intensities start to decrease with increasing pressure. This phenomenon has also been documented in the literature; yet, a comprehensive understanding of its underlying causes remains elusive [11]. The change in reflection spectra upon applied pressure (Figure 2a) was measured along with the releasing pressure (Figure 2b). Both measurements show almost negligible signs of hysteresis and the complete reproducibility of data (Figure 2c). Also, a fast response time of 10 s was recorded for all measurements.

**Figure 3:**
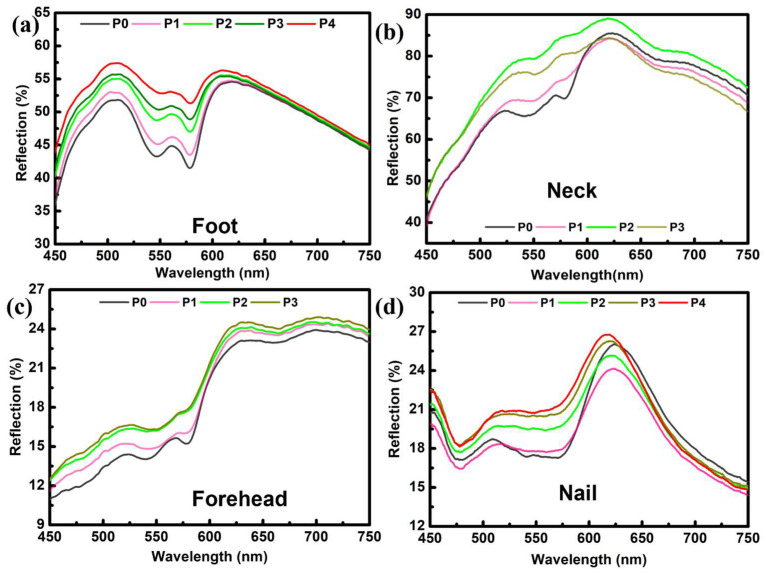
(**a**) Reflection spectra of foot. The lowermost curve (black) represents the “no pressure” and the uppermost (red) curve represents the higher-pressure P4. (**b**) Reflection spectra of the neck with increasing pressure from zero pressure to P3. (**c**) Reflection spectra under applied pressure on the forehead. (**d**). Reflection spectra of the nail, measured under different pressures.

The reflection spectroscopy of the foot is depicted in Figure 3a. Three distinct sites were selected for pressure administration and the subsequent measurement of the reflection spectra. Two sites were chosen along the dorsal (upper) surface of the foot, and one point was picked on the plantar (lower) surface. A series of probe pressures were exerted, and spectroscopic data were collected for each pressure level. The precise measurement of probe pressure remained uncertain due to inadequate provisions for pressure recording. Therefore, the optical fibers probe was just pressed against the foot to exert different pressures (P0–P4). Figure 3a displays the reflection spectra measured for a range of pressures (P0 to P4). There is a noticeable difference in the magnitude of reflected light when compared with finger spectra. This phenomenon can also be correlated with tissue morphology at a specific place. Even at the largest pressure of P4, the presence of the “W” shape is evident, suggesting that further pressure can be applied until this peak is transformed into deoxyhemoglobin.

The clinical significance of neck and forehead skin lies in their high prevalence as sites for the development of malignant lesions. Each tissue exhibits a unique site-specific tissue morphology, resulting in distinct reflection spectra among various tissues. Again, due to the limitation of the experimental set for the quantification of pressure values, the optical-fiber probe was pressed against the skin of volunteers’ necks and forearms to ensure increasing pressure in the order of P1–P3. Figure 3b illustrates a noticeable rise in reflection for P1 and P2, followed by a subsequent decline in reflection for P3. The reflection spectra may exhibit variations depending on the specific tissue morphology. Therefore, we formulated a hypothesis regarding the presence of a site-specific effect. The expansion of compressed arteries leading to a reduction in reflection is highly improbable. However, this phenomenon occasionally occurs due to the compression of tissue, resulting in the penetration of light to deeper layers where substantial blood veins are situated. This phenomenon has the potential to enhance the absorption of oxyhemoglobin and concurrently reduce the level of reflection [12]. Conversely, the reflections exhibited a positive correlation with elevated pressure applied to the forehead, indicating a reduction in the level of oxyhemoglobin absorption (Figure 3c). Under increasing pressure, the distinctive “W” shape gradually diminished and became more flattened, serving as an indicator of the presence of the deoxyhemoglobin peak.

Figure 3d displays nail reflection spectra. The load was applied with increments of 100 g for each measurement, and pressure against that load was calculated using the pressure formula. The nail, being made of a stronger substance [23], can withstand greater pressure than the skin. These readings were taken using a probe pressure of 0–390 kPa. Figure 3d displays the typical characteristic trend associated with the signature peak of oxyhemoglobin. Compared to the previous plots, this shape was extended over a more extensive range of wavelengths (475–575 nm). The different morphology of the nail to the skin explains this change. A usual trend of decreasing absorption with increasing pressure can be seen from the plots. Measuring the reflection spectra of the nail revealed that the flattening of the “W” shape with increasing pressure was very weak, indicating that a great deal of pressure is needed to obtain the deoxygenated peak.

**Figure 4:**
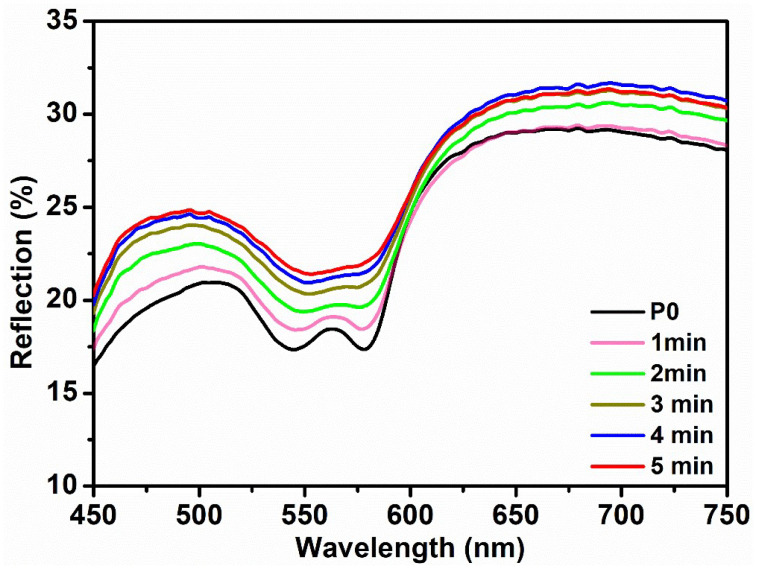
Time-based reflection spectra at a contact pressure applied on the upper part of the arm. Measurements were taken with a time interval of 1 min.

The pressure-based DRS measurements were taken for the forearm using the same optical probe and pressing it against the skin to apply different pressure. However, the efficacy of this approach remains uncertain, and the quantification of probe pressure is unattainable. Therefore, the blood occlusion method was used to reduce the blood flow and to record reflection spectra at different times. A pressure cuff was administered to the upper arm at a bearable pressure to fulfill its intended objective. The initial spectra were acquired prior to the administration of the pressure cuff (Figure 4—black curve). Subsequently, a pressure cuff was administered, and reflection spectra were measured at 1 min intervals. An apparent increase in the reflection spectra in the range of 540 nm to 570 nm can be seen in the plots. The presence of a pressure cuff led to a reduction in blood flow in the forearm, resulting in the decreased absorption of oxyhemoglobin. Hence, Figure 4 exhibits a noticeable elevation in the intensity of the reflection. Also, with increasing time, the flattering of the “W” shape became visible, indicating the conversion of the oxyhemoglobin peak to deoxyhemoglobin [24]. This suggests that the prolonged use of a pressure cuff brings discomfort and may result in the development of serious health conditions. 

**Figure 5:**
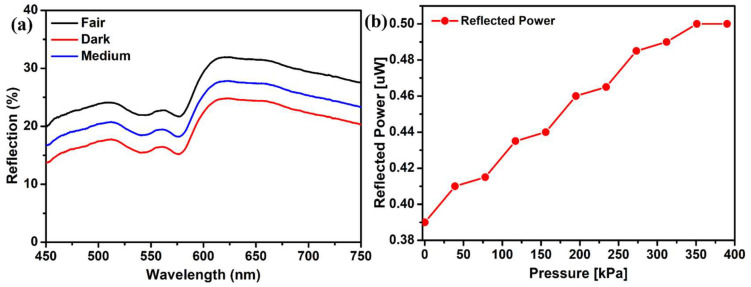
(**a**) Reflection spectra of different skin-tone types: fair, dark, and medium. (**b**) Reflection intensity with increasing pressure using green laser. A set of pressures were applied to the left-hand index finger.

The effect of different skin tones was also analyzed using this DRS technique. Reflection spectra of volunteers’ fingertips with different skin tones, including fair, medium, and dark, were recorded. All skin types showed the characteristic peak of oxyhemoglobin at the same wavelength, with negligible differences in shape. The only difference recorded was the reflection intensities. Figure 5a shows that fair skin reflects more light than darker ones, suggesting that fair people absorb less light. Also, the reflection intensity is lower for the dark skin tone because of more absorption [25]. 

A green laser was used to elucidate the spectra since the oxyhemoglobin peak exists in the green wavelength region. The original white light source was substituted with a green laser of a wavelength of 532 nm. Also, the spectrometer was replaced with a photodetector. A series of pressures were exerted on the finger, and the corresponding alterations in the reflected light intensity were documented. Figure 5b illustrates the reflected power intensity observed at various pressure levels when employing the green laser. The plot displays a stair-like tendency as the load is uniformly varied, indicating that the change in reflection intensity is not uniform [6]. An increase in pressure beyond 350 kPa does not change the reflected power intensity, showing that the muscle can no longer contract. An increase, with the increasing pressure, in the reflection intensity can be seen, which verifies the previous experimental results obtained with white light.

## 4. Discussion

The presented research demonstrates the utility of diffusive reflection spectroscopy (DRS) as a valuable tool for non-invasive dermal studies, particularly in assessing the influence of probe pressure on various human body parts. DRS measurements showed a clear “W”-shape trend in the visible wavelength range, representing the oxyhemoglobin level on that specific site. The consistent “W”-shape trend observed in the DRS spectra, particularly in the wavelength range between 500 and 600 nm, underscores the sensitivity of this technique to changes in probe pressure. An increase in the reflection spectra intensities was observed with increasing pressure. These results significantly agree with the previously published literature [1,3,9,11,13,18].

The effect of pressure on the human skin and the change observed in the reflection spectra can be understood by looking at its structure and morphology. The skin is a multifaceted tissue characterized by its heterogeneous nature, with a varying spatial distribution of blood and pigment content at different depths [1,26,27]. The skin is composed of three primary layers, namely the epidermis, dermis, and subcutaneous adipose tissue. The epidermis, which is approximately 100 µm thick, is the outermost layer and lacks blood vessels. The dermis, with a thickness ranging from 1 to 2 mm, is a vascularized layer, in which primary absorption is due to the presence of oxyhemoglobin (Figure 2d) [28,29]. The change in the reflection spectra in the 500–600 nm range is associated with the absorption of oxyhemoglobin. An elevated pressure or load exerts an inhibitory effect on the upper dermal blood network and the capillary loops [9,12]. This phenomenon leads to an augmentation of reflection within this specific band of wavelengths. To clarify, applying greater pressure to the fingertip and other parts results in the outward displacement of blood and a subsequent reduction in the overall blood volume beneath the probe. Consequently, this drop in blood content yields a diminished quantity of oxyhemoglobin (Figure 2d). Consequently, the reduction in the absorption of oxyhemoglobin results in an elevation in the intensity of reflection within this particular region [9,11,12,21].

An important aspect of this study was the investigation of different skin tones including dark, medium, and fair. The inclusion of a diverse set of participants underscores the relevance of these findings across various populations. It is worth noting that skin tone can influence the baseline optical properties of the skin, which, in turn, affect the observed spectral changes under varying probe pressures [17,30]. Further research could delve deeper into how skin tone impacts these optical properties. An additional layer of verification was provided using a green laser light source (532 nm) to verify the observed rise in reflection intensity with increasing probe pressure. The observed stair-like increase in the intensity of reflected power aligns with previously documented findings in the existing literature [9]. The fact that DRS- and laser-based measurements yielded similar findings bolsters the validity of the data and provides further evidence for the method’s potential clinical utility.

The proposed pressure-based Diffusive Reflection Spectroscopy (DRS) system has vast potential across various applications that involve monitoring pressure on the human body in response to varying pressures. In the realm of sports and athletics, it offers athletes invaluable insights into factors like muscle fatigue, joint stress, and circulation changes, optimizing training and reducing injury risks. Additionally, DRS presents opportunities for injury prevention through the early detection of high-pressure areas, benefiting athletes and weightlifters alike. Beyond sports performance, this technology has applications in exercise science, sports medicine, orthopedics, and wearables, paving the way for innovative pressure-sensing devices and diagnostic tools. This research opens doors to transformative advancements in healthcare, biomechanics, and diverse fields by comprehensively understanding skin reactions to varied pressures, promising real-time, non-invasive pressure monitoring capabilities.

## 5. Conclusions

A non-invasive comprehensive dermal study was performed under the influence of different probe pressures for various human body parts, including the fingertip, foot, neck, forehead, forearm, and nail. Diffuse reflection spectroscopy was employed to study the influence of probe pressure. The measured DRS spectra showed a clear “W”-shape trend associated with the signature oxyhemoglobin peak in the visible wavelength range between 500 and 600 nm. It was observed that the size and shape of the peak varies for different examination sites because of the difference in tissue composition and morphology. The change in the reflection spectra was also observed with increasing probe pressure. The blood content under the examination site reduces with increasing probe pressure, which gives rise to the increase in reflection spectra because of reduced absorption due to oxyhemoglobin in the region under consideration. The same results were obtained using green laser, which showed an increased reflected power with increasing probe pressure. The effect of different skin tones was also studied using DRS measurements. Additionally, it was discovered that pressure-based DRS does not adhere to a constant pattern. One of the major challenges in this study was the quantification of pressure values for different body parts. In conclusion, leveraging diffusive reflection spectroscopy, this comprehensive dermal study provides valuable insights into the interplay between the probe pressure and skin optical properties. The findings offer a foundation for further research and the development of practical applications, highlighting the potential for this technique to contribute to the diverse fields of medicine, sports science, and beyond.

## Figures and Tables

**Table 1:** Pressure calculation against every load.

Pressure	Weight [g]	Area [mm^2^]	Force [N]	Pressure [kPa]
P1	50	12.56	0.49	39
P2	100		0.98	78
P3	150		1.48	117
P4	200		1.96	156
P5	250		2.45	195
P6	300		2.94	235

## Data Availability

Data is available upon request from the corresponding authors.
